# Extent of induced abortions and occurrence of complications in Kinshasa, Democratic Republic of the Congo

**DOI:** 10.1186/s12978-019-0727-4

**Published:** 2019-05-08

**Authors:** Daniel Katuashi Ishoso, Antoinette Kitoto Tshefu, Thérèse Delvaux, Yves Coppieters

**Affiliations:** 1 Community Health Department, Kinshasa School of Public Health, University of Kinshasa, PO Box11850, Kinshasa 1, Democratic Republic of Congo; 2 Public Health Department, Institute of Tropical Medicine, Antwerpen ITM, Antwerpen, Belgium; 3 Research Centre “Policies and Health Systems - International Health”, School of Public Health, Université libre de Bruxelles (ULB), Brussel, Belgium

**Keywords:** Democratic Republic of the Congo, Induced abortions, Incidence

## Abstract

**Background:**

Due to a lack of relevant data on induced abortions in the Democratic Republic of the Congo (DRC) as well as the persistence of maternal deaths in the country, this study aims to analyse the extent of induced abortions and occurrence of complications in Kinshasa.

**Methodology:**

This cross-sectional study was conducted with a sample of 460 women who were interviewed about their experiences as females, and provided information of 1444 women of childbearing age living in Kinshasa. Respondents’ households were selected to represent the five types of residential quarters in Kinshasa, differentiated by cultural, socioeconomic, and infrastructural characteristics. Information was collected using a survey form and analyzed.

**Results:**

Among all confidantes included in the study, 5.5% (95% CI: 4.4–6.8%) had induced abortions during 2015, a rate of 55.0 abortions per 1000 women of childbearing age. This practice was significantly performed amongst single/separated/divorced women; those without formal education, or primary-school education, and women who consumed excessive alcohol. Most abortions were induced by the administration of high doses of medication, by the women themselves or by health workers. A percentage of 51.9% (95%CI: 40.4–63.3%) of induced abortions led to complications, which were predominantly haemorrhagic. Moreover, 39% of patients had a complication for which they sought care, and of whom 12.5% had genital trauma or uterine perforation/intestinal necrosis.

**Conclusion:**

Induced abortion is a public health problem in Kinshasa due to its frequency of practice, the complications that occur, and the absence of major surgeries in the health care package offered by the health centres or dispensaries that also provide the treatment of some serious complications. Thus, there is a need to focus on the enhancement of the health care package offered by health centres to include appropriate measures in favour of maternal health.

## Plain English summary

Due to a lack of relevant data on induced abortions in the Democratic Republic of the Congo (DRC) as well as the high rate of maternal deaths, this study aims to analyse the extent of induced abortions and occurrence of complications in Kinshasa.

To achieve this, 460 women were interviewed about their experience, and provided information about 1444 women of childbearing age living in Kinshasa.

Among all confidantes included in the study, 5.5% had induced abortions during 2015, a rate of 55.0 abortions per 1000 women of childbearing age in Kinshasa. This practice was significantly higher in single/separated/divorced women, those without a formal education or primary-school education, and women who consumed excessive alcohol. Most abortions were induced by the administration of high doses of medication, by the women themselves or by health workers. 51.9% (95% CI: 40.4–63.3%) of induced abortions led to complications, that were predominantly haemorrhagic. Moreover, 39% of patients with complications went to a health centre or dispensary to obtain care of whom 12.5% had genital trauma or uterine perforation/intestinal necrosis.

In conclusion, induced abortion is a public health problem in Kinshasa due to its frequency of practice, the complications that occur, and the absence of major surgeries in the health care package offered by the health centres or dispensaries that also provide the treatment of some serious complications. Thus, there is a need to focus on their prevention and the enhancement of the health care package offered by health centres to include appropriate measures in favour of maternal health.

## Article summary

### Strengths

The first survey that estimated directly from the community the frequency of induced abortion and occurrence of complications in Kinshasa, Democratic Republic of the Congo.

### Limitation

The possibility that the extent of induced abortion and its consequences was underestimated because the information was collected from non-primary sources; respondents could report only what they knew about their confidantes and were willing to report.

## Background

Annually, about 25 million unsafe abortions were estimated to have taken place worldwide [1], the majority of these occurring in developing countries, where about 7 million women are admitted to hospitals due to complications [2].

Complications from unsafe abortions account for about 7.9% (4.7–13.2%) of maternal deaths [3]. In addition to maternal deaths, high morbidity can be observed long-term such as premature births, psychiatric sequelae, infertility or subfertility, chronic pelvic pain, ectopic pregnancy, and spontaneous abortions [4–7].

An essential component in continuing efforts to reduce the incidence and consequences of unsafe abortions remains empirical evidence of the magnitude of the problem. Data of this nature may also be useful for assessing the impact of investments made to lessen these consequences. Studies to estimate the incidence of unsafe abortions in most developing countries where access to safe medical abortion is severely limited are difficult to conduct properly due to the clandestine nature of this phenomenon.

One of the estimation methods used in these countries is the, “Abortion Incidence Complications Method” (AICM), which is an indirect estimation method. According to this approach, the incidence of abortion is calculated as the sum of all abortions that resulted in complications treated or untreated in a health facility, and those without complications. All these calculation elements are estimated from information collected in health facilities and from health professionals [8]. The limits of this method include the following: underestimating the incidence of abortions because it does not include cases of complications that are not treated in health facilities, permission from the health authorities to measure cases of postabortion care in their health establishments, and the tendency for providers to under-report postabortion care for fear of losing their medical license or going to jail. In view of these limits, Anonymous Third Party Reporting (ATPR) method can be a good alternative because it estimates directly from the community in countries where access to abortion services is highly restricted and the practice is clandestine [9].

In the Democratic Republic of the Congo (DRC), one of the developing countries where almost all induced abortions are backstreet abortions and unsafe because the restrictive laws, the prevalence of modern contraception is low at 8% [10], a situation which undoubtedly have effect on the rates of unwanted pregnancies and induced abortions. Available data on unwanted pregnancies and induced abortions are estimated on the basis of the AICM. Given the different limits of this method and the implementation of the post-abortion care strategy under way in this country, this study proposes to use the confidante approach, an ATPR method, which entails surveying women about their confidantes’ abortions. This approach estimates directly from the community to determine, in the city of Kinshasa DRC: (i) the proportion of induced abortions amongst women of childbearing age as well as their characteristics; (ii) the determinants of induced abortions; and (iii) the frequency of post-abortion complications occurring in women of childbearing age as well as their characteristics.

## Methodology

### Study design

During March 2016, a cross-sectional study was conducted in households in Kinshasa, the capital of the DRC. Kinshasa was chosen as the study setting because induced abortion occurs predominantly in cities [11–13].

### Study population

The study population included in the study were women of childbearing age living in households in the city of Kinshasa and who confided secret and/or personal events of their lives to interviewees.

### Confidante approach and sampling

The confidante approach used is an ATPR method, which involves gathering information from randomly selected respondents about people (friends, sisters, colleagues, etc.) who entrust them with secret events of their lives (confidants). Respondents thus generated the sample without being part of it [9, 14, 15].

In this study, respondents were women of childbearing age, the sample generated was also women of childbearing age.

It should be noted that the respondents from whom was generated the sample were selected randomly and in a representative way from the city of Kinshasa, and the sample generated from these respondents consists essentially of their confidants. The respondents were not part of the sample and their personal information was not considered [9].

Thus, the first step to implementing the ATPR method was to draw a representative sample of respondents [9].

Random selection of respondents interviewed:

For the random selection of respondents, the sample size was calculated using the formula “the square of 1.96 which is the value of the standard normal distribution coefficient corresponding to a significance level of alpha of 0.05, multiplied by the proportion of women of childbearing age in the city of Kinshasa (we used the proportion of 50 % to have the highest minimum size), multiplied by its complement (1-0.50), divided by the square of precision degree that we assumed to be at 5 % too” The minimal sample size calculated was 384 women of childbearing age. We added 20% (*n* = 76) to the sample to account for non-respondents, yielding a total sample of 460Moreover, to approach the respondents in a representative way of the city of Kinshasa, five strata of the health zones (the basic operational level of the DRC’s health system) of this city were formed according to their cultural, socio-economic and infrastructural similarities [16]; then, from each stratum, a health zone was randomly drawn, so that a total of five health zones representative of the city of Kinshasa were surveyed. The sample of 460 respondents was distributed amongst these five health zones proportionally to the size of their respective populations. The size of different populations was given by the central offices of the respective health zones, from the projection of 1982 census data.

In each health zone, all health areas were drawn, the sample of the health zone was distributed amongst these health areas proportionally to the size of their respective populations. In each health area three avenues were randomly drawn, the health area sample was distributed amongst these three avenues proportionally to the size of their respective populations.

In each selected avenue, a plot identification survey (parcels/residences) was conducted in which there was at least one household with at least one woman of childbearing age. The results of this survey and the number allocated to the avenue were used to calculate a poll step, which made it possible to identify the parcels (residences) to be investigated. If a parcel survey of more than one household had at least one woman of childbearing age, the household to be surveyed was chosen randomly. Similarly, in a household survey where there were several women of childbearing age, one woman was chosen randomly and interviewed about her confidantes.

### Data collection, information sources, and variables

Professional health investigators collected data using a survey form, asking respondents to recall the characteristics of induced abortion for their confidents during the year 2015.

Information on induced abortion was collected as any other characteristic of the confidante, and its difference with spontaneous abortion was ensured at the time of the interview by reminding the respondent of what it was, because the population of Kinshasa hardly confuse these two concepts even in the local language (“alongola zembi” for induced abortion and “zemi elongwaki” for spontaneous abortion). Those of excessive alcohol use (more than two standard drinks per day or driving under the influence of alcohol) was collected because of the potential effects of alcohol on self-control and subsequent reckless behaviour, such as unprotected sex, which can cause unwanted pregnancy and induced abortion [17].

As for the variable age, the group of adolescents consisted of any subject aged 19 years or younger, and this was in line with the definition provided by UNICEF, UNAIDS, and WHO.

### Data processing and statistical analysis

Data were entered into an Epi Info software program version 3.5.4, exported to Microsoft Excel, and analysed using Statistical Package for the Social Sciences (SPSS) version 20, IBM.

The non-probabilistic nature of the sampling prevented inferences from been drawn about the proportion of women of childbearing age according to the types of district in Kinshasa, but rather of those relating to the sociodemographic and clinical characteristics (including the history of induced abortion) [18].

The rate of induced abortion expressed per 1000 women of childbearing age in 2015 was calculated by the ratio of induced abortions to person-year 2015 (the number of respondent’s confidants in 2015 in the denominator and the induced abortions performed among these confidants in the numerator).

Categorical variables were reported as a frequency and percentage, and groups were compared using the χ2 test. The forward stepwise logistic regression helped to identify independent predictors of induced abortion, complications thereof, and referral hospital use in cases of complication. Only variables associated with induced abortion, and complications thereof in the bivariate analysis were included in the final model. The odds ratio (OR) with a corresponding 95% confidence interval was reported to quantify the strength of association. Significance was set at *p*-value of less than 0.05.

### Ethical considerations

The National Ethics Committee of the Kinshasa School of Public Health, University of Kinshasa, approved this study before the collection of data (Ethics Certificate N^°^ ESP/CE/015/2016). Authorisation was also provided by health and politico-administrative authorities. The informed consent of women (respondents) was requested systematically, and the confidentiality of women of childbearing age was respected to the maximum extent possible by imposing anonymity.

## Results

A total of 460 respondents reported on 1444 confidents, with a median of 3 (IQR:2–4) confidents.

### Frequency of induced abortions (Table 1)

During 2015, amongst 1444 confidantes, a percentage of 5.5% (95% CI: 4.4–6.8%) had induced abortions, which corresponds to the rate of 55.0 abortions per 1000 women of childbearing age in Kinshasa (95% CI: 44.0–68.0%).

**Table 1:** Frequency of induced abortion in 2015

Confidante *n* = 1444	n’ (%)	CI 95%
Induced abortion		
Yes	79 (5.5)	4.4 to 6.8
No	1365 (94.5)	93.2 to 95.6
Total	1444 (100)	

With n = number of subjects in the sample; n’ = number of subjects in the sample subgroups

### Characteristics of induced abortions (Tables 2 and 3)

The median age (IQR) of the confidante’s population was 27 years (22–32); almost half of the women were single or separated or divorced or widowed and had completed secondary school. They had at least one child and most of them had no income. Most confidantes had neither a history of induced abortion and nor did they consume tobacco or alcohol.

**Table 2:** Descriptive analysis

Variables	n’ (%)	Median (P25-P75)
Number of confidants per respondent (*n* = 460)		3 (2–4)
Confidante’s residence (*n* = 1444)		
Quarters of old cities	285 (19.7)	
Quarters of planned cities	307 (21.3)	
Eccentric quarters and of extension	403 (27.9)	
Residential quarters	171 (11.8)	
Semi-rural quarters	278 (19.3)	
Total	1444 (100)	
Adolescence (years) (*n* = 1444)		27 (22–32)
Yes	243 (16.8)	
No	1201 (83.2)	
Total	1444 (100)	
Level of education of the confidante (*n* = 1444)		
None	28 (1.9)	
Primary	305 (21.1)	
Secondary	710 (49.2)	
High	401 (27.8)	
Total	1444 (100)	
Employment of the confidante (1442)		
State Employee	145 (10.1)	
Private employee	147 (10.2)	
Self-employed	360 (25.0)	
Student	341 (23.6)	
Unemployed	449 (31.1)	
Total	1442 (100)	
Income of the confidante (1442)		
No income	652 (54.7)	
Worker	790 (45.3)	
Total	1442 (100)	
Marital status (*n* = 1443)		
Single/separated/divorced/widowed	886 (61.4)	
Married/cohabiting	557 (38.6)	
Total	1443 (100)	
Confidante parity (*n* = 1444)		
Nulliparous	686 (47.5)	
Primiparous/Multiparous	758 (52.5)	
Total	1444 100)	
Excessive use of alcohol (*n* = 1444)		
Yes	504 (34.9)	
No	940 (65.1)	
Total	1444 (100)	
Use of tobacco (*n* = 1444)		
Yes	20 (1.4)	
No	1424 (98.6)	
Total	1444 (100)	
Confidante abortion background (*n* = 1444)		
No abortion background	1191 (82.5)	
One or several abortions	253 (17.5)	
Total	1444 (100)	

With: n = number of subjects in the sample; n’ = number of subjects in the sample subgroups; SD = Standard Deviation

The eccentric quarters and of extension are essentially unplanned building. They are isolated, unregistered and mostly inhabited by low income social strata. Accessibility is at random and impractical in some places. Pedestrian mobility is important. Public infrastructures are almost non-existent. The public transportation means are uncertain and, pedestrian accessibility is difficult and not fitted

**Table 3:** Characteristics of induced abortion

Variables	n’ (%)
Abortionists (*n* = 79)	
Health and related worker	32 (40.5)
Confidante herself	40 (50.6)
Traditional healer	7 (8.9)
Total	79 (100)
Method used (*n* = 79)	
Curettage/Aspiration	25 (31.6)
Injections	6 (7.6)
High doses of drugs	40 (50.6)
Potion/syrup/purge	4 (5.1)
Agent caustic/Stems/other	4 (5.1)
Total	79 (100)

High doses of drugs were administered orally

Most of these induced abortions were performed after the administration of high doses of drugs by the women themselves (50.6%) or by health workers (40.5%).

### Determinants of induced abortion (Table 4)

Multivariate analysis shows that: single or separated or divorced women or widows had significantly a higher odds of having an induced abortion than married women (Adjusted OR = 2.7; 95% CI: 1.4–4.9); women without formal education or primary-school education had significantly a higher odds than women with secondary education (Adjusted OR = 2.2.; 95% CI: 1.1–4.8); and women who consumed excessive alcohol had a significantly higher odds than those who did not (Adjusted OR = 3.2; 95% CI: 2.2–6.9).

**Table 4:** Determinants of induced abortion (bivariate and multivariate analysis)

	Induced abortion			
	Bivariate analysis		Multivariate analysis (LR)	
Independent variables	Crude OR (CI95%)	p	Adjusted OR (CI95%)	p
Adolescence (≤ to 19 yrs)		NS		
Yes (n’ = 243)	1.6 (0.9–2.7)			
No (n’ = 1201)	1			
Patient’s residence		NS		
Quarters of old cities (n’ = 285)	1.8 (0.8–4.1)			
Eccentric quarters and of extension (n’ = 403)	1.6 (0.8–3.7)			
Residential quarters (n’ = 171)	2.1 (0.8–5.0)			
Semi-rural quarters (n’ = 278)	2.4 (1.1–5.4)			
Quarters of planned cities (n’ = 307)	1			
Level of education of the confidante		0.005		0.044
None/Primary (n’ = 333)	2.9 (1.5–6.2)		2.2 (1.1-4.8)	
Secondary (n’ = 710)	1.9 (0.9–3.8)			
High (n’ = 401)	1		1	
Income of the confidante		NS		
No income (n’ = 790)	1.3 (0.8–2.1)			
Worker (n’ = 652)	1			
Marital status of the confidante		0.010		0.002
Single/separated/divorced/widowed (n’ = 842)	1.9 (1.2–3.2)		2.7 (1.4 4.9)	
Married/cohabiting (n’ = 601)	1		1	
Parity of the confidante		NS		
Nulliparous (n’ = 685)	1.0 (0.6–1.5)			
Primiparous/Multiparous (n’ = 758)	1			
Abortion background of the confidante		NS		
One or several abortions (n’ = 1191)	1.3 (0.7–2.7)			
No abortion background (n’ = 253)	1			
Excessive use of alcohol		< 0.001		< 0.001
Yes (n’ = 504)	2.8 (1.7–4.4)		3.2 (2.2–6.9)	
No (n’ = 940)	1		1	
Use of tobacco by the confidante		NS		
Yes (n’ = 20)	1.9 (0.4–8.5)			
No (n’ = 1424)	1			

With: OR = Odds Ratio; P = p-value; LR = logistic regression

### Frequency and characteristics of induced abortion-related complications (Table 5)

A percentage of 51.9% (95% CI: 40.4–63.3%) of induced abortions led to complications, that were predominantly haemorrhagic, and occurred in eccentric and semi-rural districts of Kinshasa. Moreover, there was a percentage of 39% of patients with complications who went to a health centre or dispensary to obtain care and of whom 12.5% had genital trauma or uterine perforation/intestinal necrosis.

**Table 5:** Frequency and characteristics of complication due to induced abortion

Induced abortion n = 79	n’ (%)	CI 95%
Induced abortion-related complication		
Yes	41 (51.9)	40.7 to 63.3
No	38 (48.1)	36.7 to 59.6
Total	79 (100)	
Induced abortion-related complication *n* = 41	n’ (%)	
Type of complication		
Haemorrhage	27 (65.8)	
Infection	6 (14.6)	
Genital trauma	4 (9.8)	
Uterine perforation/Intestinal necrosis	4 (9.8)	
Total	41 (100)	
Residence of complication cases		
Quarters of old cities	7 (17.1)	
Eccentric quarters and of extension	15 (36.6)	
Residential quarters	5 (12.2)	
Semi-rural quarters	9 (21.9)	
Quarters of planned cities	5 (12.2)	
Total	41 (100)	
Type of solicited health facility if complication		
Tertiary facility	8 (19.5)	
Public secondary facility	6 (14.6)	
Private secondary facility	9 (22.0)	
Health center or Dispensary	16 (39.0)	
No precision	2 (4.9)	
Total	41 (100)	
Type of complication visited health center or dispensary for care		
Haemorrhage	12 (75.0)	
Infection	2 (12.5)	
Genital trauma or uterine perforation/Intestinal necrosis	2 (12.5)	
Total	16 (100)	

## Discussion

In the study 5.5% of confidantes had induced abortions, which corresponds to the rate of 55.0 abortions per 1000 women of childbearing age. The findings of this study reflect the poor success rate of interventions implemented in family planning settings. The aim of these interventions is to reduce the need to resort to induced abortion by reducing the rate of unwanted pregnancy, mainly through the use of modern contraception [19]. Nationally, since 2007, the prevalence of modern contraceptive use in the DRC is low, at around 8% [10].

The rate of induced abortion in the DRC is much higher compared with other African countries such as Malawi (46.1 per 1000 women of childbearing age), Tanzania (34.4 per 1000 women of childbearing age) and Rwanda (51.6 per 1000 women of childbearing age) where the rates of modern contraceptive use are higher [20].

A percentage of abortions (50.6%) were self-induced by women of childbearing age, and 40.5% were induced by health workers or simulators. Most methods used were medicinal. In the DRC and other developing countries, regulations on the sale and use of pharmaceutical products are scarcely observed, and the practices of self-medication and diversification of remedies are commonplace; they constitute the therapeutic choices of the majority of persons before they resort to a sanitary structure [21–23].

The researchers found a significant correlation between excessive alcohol use (more than two standard drinks per day or driving under the influence) and the incidence of induced abortion. Such consumption may directly affect the decision to abort, or lead to unprotected intercourse, which could result in unwanted pregnancy [17]. Moreover, in the present study a significant association was observed between single or separated or divorced women or widows without formal education or primary-school education, these results corroborating those reported by various authors [11, 12, 24–28]; and can be understood within the social context of Kinshasa as pregnancy out of wedlock is not tolerated (thus impelling unmarried pregnant women to abort when the fathers are not ready for marriage) and the exclusion of sex education in the primary school curriculum.

During 2015, a percentage of 51.9% of the induced abortions performed led to complications. The high incidences of these complications reflect upstream a serious problem to perform these abortions in Kinshasa. This situation is very similar to those in other countries, such as Rwanda and Burkina Faso, where 40% of induced abortions led to complications [29, 30]. Most complications occurred in eccentric and semi-rural districts of Kinshasa, which can be explained by a low socioeconomic level which limits women in their purchase of medicines for post-abortion care as well as the poor quality of the abortion procedures by abortion providers in these neighbourhoods (poor quality of the procedures). Haemorrhage was the predominant type of complication, followed by infection and genital trauma. This finding is in agreement with those of many other studies conducted in similar contexts [31–33].

During 2015, 39% of confidents with post-abortion complications visited a health centre or dispensary for care. Of these, there were cases of genital trauma and uterine perforation complication that should be treated at the referral health facilities that offer the full package of care. The health centre or dispensary that provided treatment for them has a mandate only to offer the minimal package of ambulatory care that does not include major surgical procedures such as laparotomy to repair a uterine perforation. This situation can be explained by a greater facility, financial and geographical access to a health centre or dispensary than to referral hospitals. This facility makes the health centre or dispensary the most widely attended health infrastructure in both Kinshasa (67.5%) and nationally (63.2%) [34].

It should be noted the possibility that the extent of induced abortion and its consequences were underestimated because the information was collected from non-primary sources, in a geographical context where induced abortion is illegal and poorly perceived by the population; respondents could only report what they knew about their confidents and what they felt safe reporting to researchers.

## Conclusion

This study confirmed that induced abortion is a public health problem in Kinshasa due to the frequency of the practice, the complications that occur, and the absence of major surgeries such as laparotomy in the health care package offered by the health centres or dispensaries that also provide the treatment of some serious complications.

In the context of the DRC, where the prevalence of modern contraceptive use is low despite the efforts of the Ministry of Public Health in raising awareness and distributing modern contraceptives through the national reproductive health program supported by several partners and laws prohibit induced abortion, leading to its clandestine practice with serious complications, the analysis of the management of induced abortion-related complications in the health structures and that of possibility of extending the health care package offered by health centres to major surgeries are urgently needed to offer appropriate measures in favour of maternal health.

## Abbreviations

CI: Confidence interval
DRC: Democratic Republic of the Congo
IQR: InterQuartile Range
n: Number of subjects in the sample
n’: Number of subjects in the sample subgroups
OR: Odds Ratio
p: *p*-value
RDS: Respondent Driven Sampling
SD: Standard Deviation
SPSS: Statistical Package for the Social Sciences
WHO: World Health Organization

## References

[1] Ganatra B, Gerdts C, Rossier C, Johnson BR Jr, Tuncalp Ö, Assifi A, Sedgh G, Singh S, Bankole A, Popinchalk A, Bearak J, Kang Z, Alkema L. Global, regional, and subregional classification of abortions by safety, 2010–14: estimates from a Bayesian hierarchical model. Lancet. 2017. 10.1016/S0140-6736(17)31794-4 PMC5711001 28964589

[2] Singh S, Maddow-Zimet I (2015). Facility-based treatment for medical complications resulting from unsafe pregnancy termination in the developing world, 2012: a review of evidence from 26 countries. BJOG: An International Journal of Obstetrics & Gynaecology.

[3] Say L, Chou D, Gemmill A, Tunçalp Ö, Moller AB, Daniels J, Gülmezoglu AM, Temmerman M, Alkema L (2014). Global causes of maternal death: a WHO systematic analysis. Lancet Glob Health.

[4] Thorp JM, Hartmann KE, Shadigian E (2003). Long-term physical and psychological health consequences of induced abortion: review of the evidence. Obstet Gynecol Surv.

[5] Moreau C, Kaminski M, Ancel PY, Bouyer J, Escande B, Thiriez G, Boulot P, Fresson J, Arnaud C, Subtil D, Marpeau L, Rozé JC, Maillard F, Larroque B (2005). Previous induced abortions and the risk of very preterm delivery: results of the EPIPAGE study. EPIPAGE Group BJOG.

[6] Major B, Cozzarelli C, Cooper ML, Zubek J, Richards C, Wilhite M, Gramzow RH (2000). Psychological responses of women after first-trimester abortion. Arch Gen Psychiatry.

[7] De Tourris H., Henrion R., Delecour M. Abrégé de Gymécologie et obstétrique. 6ème édition. Paris Milan Barcelone: Masson 1994: 671.

[8] Singh S, Prada E, Juarez F, The abortion incidence complications method: a quantitative technique, dans: Singh S, Remez L et Tartaglione A, eds., Methodologies for Estimating Abortion Incidence and Abortion-Related Morbidity: A Review, New York: Guttmacher institute; et Paris: International Union for the Scientific Study of Population, 2010, pp. 71–98.

[9] Rossier C, Singh S, Remez L, Tartiglione A (2010). The anonymous third party reporting method. Methodologies for estimating abortion incidence and abortion-related morbidity: a review.

[10] Ministère du Plan et Suivi de la Mise en œuvre de la Révolution de la Modernité (MPSMRM), Ministère de la Santé Publique (MSP), ICF International (2014). Enquête démographique et de santé en République Démocratique du Congo 2013–2014.

[11] Ibrahim IA, Onwudiegwu U (2012). Sociodemographic determinants of complicated unsafe abortions in a semi-urban Nigerian town: a four-year review. West Indian Med J.

[12] Ekanem EI, Etuk SJ, Ekabua JE, Iklaki C (2009). Clinical presentation and complications in patients with unsafe abortions in University of Calabar Teaching Hospital, Calabar, Nigeria. Niger J Med.

[13] Kinoti SN, Gaffikin L, Benson J (2004). How research can affect policy and programme advocacy: example from a three-country study on abortion complications in sub-Saharan Africa. East Afr Med J.

[14] Sedgh G, Rossier C, Kaboré I, Bankole A, Mikulich M (2011). Estimating abortion incidence in Burkina Faso using two methodologies. Stud Fam Plan.

[15] Rossier C, Guiella G, Ouédraogo A, Thiéba B (2006). Estimating clandestine abortion with the confidants method--results from Ouagadougou, Burkina Faso. Soc Sci Med.

[16] Lelo NF, Tshimanga MC (2004). Pauvreté urbaine à Kinshasa.

[17] Organisation Mondiale de la Santé (2016). Santé des adolescentes: Risques sanitaires et solutions. Rapport final.

[18] Wilhelm M (2014). Echantillonnage boule de neige : La méthode de sondage déterminé par les répondants.

[19] Organisation Mondiale de la Santé (2015). Santé sexuelle et reproductive : Promouvoir la planification familiale. Rapport final.

[20] Donna C, Toshiko K (2013). Fiche de données sur la planification familiale dans le monde.

[21] Mbutiwi IN, Lepira BF, Dramaix WM, Meert P, Malengreau M, Nseka MN, Muanda TF, Koné D (2013). L’automédication chez des patients reçus aux urgences médicales des cliniques universitaires de Kinshasa. Santé Publique.

[22] Coppieters Y, Bivort P, Madani K, Metboul M. Analyse des facteurs de la mortalité maternelle dans le sud algérien. Santé publique 2011, 23, n°5, pp.413–426. 22177707

[23] Manzambi JK, Tellier V, Bertrand F, Albert A, Reginster JY, Van BH (2000). Les déterminants du comportement de recours au centre de santé en milieu urbain africain: résultats d’une enquête de ménage menée à Kinshasa, Congo. Tropical Med Int Health.

[24] Kigbu JH, Daru PH, Ujah IA (2009). Review of maternal deaths from unsafe abortion in Jos, Nigeria. Niger J Med.

[25] Abiodun OM, Balogun OR, Adeleke NA, Farinloye EO (2013). Complications of unsafe abortion in south West Nigeria: a review of 96 cases. Afr J Med Med Sci.

[26] Mitsunaga TM, Larsen UM, Okonofua FE (2005). Risk factors for complications of induced abortions in Nigeria. J Women's Health (Larchmt).

[27] Bernabé-Ortiz A, White PJ, Carcamo CP, Hughes JP, Gonzales MA, Garcia PJ, Garnett GP, Holmes KK (2009). Clandestine induced abortion: prevalence, incidence and risk factors among women in a Latin American country. CMAJ.

[28] Levandowski BA, Pearson E, Lunguzi J, Katengeza HR (2012). Reproductive health characteristics of young Malawian women seeking post-abortion care. Afr J Reprod Health.

[29] Bankole A, Hussain R, Sedgh G, Rossier C, Kaboré I, Guiella G (2013). Grossesse non désirée et avortement provoqué au Burkina Faso: causes et conséquences.

[30] Basinga P, Moore A, Singh S, Remez L, Birungi F, Nyirazinyoye L (2013). Unintended pregnancy and induced abortion in Rwanda: causes and consequences.

[31] Dragoman M, Sheldon WR, Qureshi Z, Blum J, Winikoff B, Ganatra B (2014). WHO multicountry survey on maternal newborn Health Research network. Overview of abortion cases with severe maternal outcomes in the WHO multicountry survey on maternal and newborn health: a descriptive analysis. BJOG.

[32] World Health Organization (2011). Unsafe abortion: Global and Regional Estimates of the Incidence of Unsafe Abortion and Associated Mortality in 2008.

[33] Grimes DA, Benson J, Singh S, Romero M, Ganatra B, Okonofua FE, Shah IH (2006). Unsafe abortion: the preventable pandemic. Lancet..

[34] Programme des Nations Unies pour le Développement. Unité de lutte contre la pauvreté (2009). Rapport final sur la pauvreté et les conditions de vie des ménages de Kinshasa. Kinshasa.

